# Grid cell remapping under three-dimensional object and social landmarks detected by implantable microelectrode arrays for the medial entorhinal cortex

**DOI:** 10.1038/s41378-022-00436-5

**Published:** 2022-09-16

**Authors:** Zhaojie Xu, Fan Mo, Gucheng Yang, Penghui Fan, Yiding Wang, Botao Lu, Jingyu Xie, Yuchuan Dai, Yilin Song, Enhui He, Shihong Xu, Juntao Liu, Mixia Wang, Xinxia Cai

**Affiliations:** 1 State Key Laboratory of Transducer Technology, Aerospace Information Research Institute, Chinese Academy of Sciences, Beijing, 100190 China; 2 University of Chinese Academy of Sciences, Beijing, 100049 China

**Keywords:** Biosensors, Nanoparticles

## Abstract

Grid cells with stable hexagonal firing patterns in the medial entorhinal cortex (MEC) carry the vital function of serving as a metric for the surrounding environment. Whether this mechanism processes only spatial information or involves nonspatial information remains elusive. Here, we fabricated an MEC-shaped microelectrode array (MEA) to detect the variation in neural spikes and local field potentials of the MEC when rats forage in a square enclosure with a planar, three-dimensional object and social landmarks in sequence. The results showed that grid cells exhibited rate remapping under social conditions in which spike firing fields closer to the social landmark had a higher firing rate. Furthermore, global remapping showed that hexagonal firing patterns were rotated and scaled when the planar landmark was replaced with object and social landmarks. In addition, when grid cells were activated, the local field potentials were dominated by the theta band (5–8 Hz), and spike phase locking was observed at troughs of theta oscillations. Our results suggest the pattern separation mechanism of grid cells in which the spatial firing structure and firing rate respond to spatial and social information, respectively, which may provide new insights into how the brain creates a cognitive map.

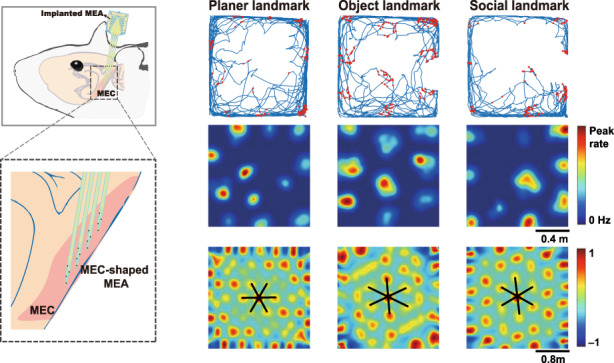

## Introduction

Spatial navigation is an essential cognitive function that is crucial to the daily life of social animals. Socially related psychiatric disorders also induce spatial cognition impairment, which seriously threatens people’s mental health^1^. Hence, understanding the neural basis supporting spatial and social behavior is of great significance. A generally accepted mechanism is that the surrounding environment is represented by cognitive maps in the brains of animals^2–4^ and humans^5,6^ with the capacity to integrate social and contextual spatial information^7,8^. The neural basis of a cognitive map is collectively formed by multiple spatially tuned cells, such as place cells^9^ in the hippocampus and grid cells^10,11^ in the MEC. Grid cells with hexagonally arranged firing fields exhibit the neural representation of the spatial environment, whereas their relationship with social information has not been reported. However, a previous study^12^ indicated that MEC cell populations revealed different responses to social and object stimuli. Whether spikes and the local field potentials (LFPs) of grid cells would exhibit similar responses and how they function according to the spatial relationship with the object and social landmarks warrants further exploration.

When animals move through environments with different visual cues, spatially tuned cells show distinct firing patterns, a function called remapping^13–15^. Two kinds of remapping were reported in which both the location and rates of firing ensembles varied or just the firing rate varied, known as global remapping or rate remapping, respectively^16,17^. However, the remapping of grid cells has not been reported in earlier studies. Grid cells were believed to provide a spatial metric reference for the spatial cognitive map^18^, and their spikes were modulated by the local theta oscillations in MEC-hippocampal circuits to maintain stable hexagonal firing patterns^19^. In traditional tests of grid cells, MEC neuronal activities and positions of freely moving animals were recorded simultaneously in a large open field with planar visual landmarks, and color changes and removal of planar landmarks were studied to indicate the invariant hexagonal patterns of grid cells^10^. However, environments with three-dimensional (3D) objects and other animals, which are more similar to the real-world setting, have rarely been used in grid cell studies. Therefore, whether 3D spatial and social information of environments would evoke novel responses of grid cells, especially remapping, is worth in-depth study.

Currently, high-density recordings of neurons in the MEC at high temporal resolution are necessary for understanding the grid-map mechanism, including spatial representation and temporal organization. Because extracellular recording is invasive, multisite detection for large numbers of neurons would inevitably increase tissue injury^20^. Compared with traditional wire electrodes, the silicon-based microelectrode array (MEA) fabricated by microelectromechanical system (MEMS) technology can increase recording sites substantially with the same amount of tissue displacement^21^. In addition, planar arrays have advantages in modification with nanomaterials to improve detection performance^22^. Moreover, multiple recording sites can be precisely geometrically arranged over a longer distance of a silicon probe, which not only allows for the simultaneous recording of neuronal activities at distinct locations but can also define the spatial relationship of isolated single neurons^23^. Importantly, the shape of silicon multishank probes can be designed for the specific brain area, which can both increase the coverage of the target area and reduce the impairment of the nontarget area^24^. Overall, MEA is a prospective tool for detecting neurophysiological signals in behavioral experiments to promote the study of neural circuit mechanisms of navigation and social behavior.

In this study, MEA designed for MEC was used in monitoring the neural activities of grid cells for freely moving rodents. The detection sites were modified with platinum nanoparticles, and the stability of the detection performance was tested. In behavioral trials, rats encountered planar, 3D object, and social landmarks in turn, and the influences of these objects and landmarks on the spatial representation of grid cells were explored at both the single-cell and population levels. On the one hand, global firing patterns and firing rate distributions of grid cells were compared under three landmarks to reveal the global remapping and rate remapping and their relationships with 3D spatial and social features in the environment. On the other hand, the theta rhythm of the LFPs and phase locking of spikes in grid cells were analyzed to illustrate the universality of theta modulation in the MEC. The results could contribute to understanding the grid-map mechanism processing spatial and social information.

## Materials and methods

### Design and fabrication of the MEA

**Fig. 1:**
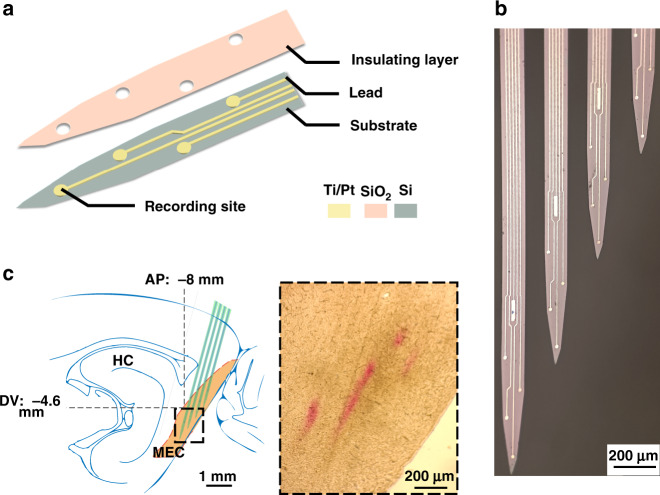
Fabrication process and shape design of the MEA. **a** Three layers, containing the insulating layer, conducting layer, and substrate, were patterned to form the structure of the MEA. **b** Optical photograph of the tip of the MEA. **c** Illustration of the arrangement of MEA probes according to the shape of the MEC (left) and a sagittal brain section with red traces of the implanted MEA (right)

In our study, an MEC-shaped four-shank MEA was designed to monitor grid cells in the MEC. Each probe was 110 μm × 30 μm (width × thickness) in cross section and separated by 90 μm. The lengths of the four shanks were 6, 5.5, 4.5, and 4 mm. Each shank contained four recording sites (diameter = 15 μm) and a reference site (15 μm × 100 μm). The spacing between sites was 150 μm (Fig. 1b). The MEA was designed to match the anatomy of the MEC, which has a maximum width of 1 mm in the sagittal plane, with a depth from 2 to 6 mm and an incline of 35°, as shown in Fig. 1c. The dimension parameters of MEA were selected to match the MEC. The overall width was 0.9 mm, and the tips of the shanks were separated by 500 μm longitudinally and 100 μm laterally, forming a tilt angle of 20°. The MEA was implanted with an angle of 15°, which not only matched the incline of MEC but also allowed for a greater moving range from the top to the bottom.

The MEA was fabricated using MEMS technology in a superclean laboratory, as previously reported^25,26^. Three layers were patterned onto a silicon-on-insulator (SOI) substrate to form the electrode structure (Fig. 1a). The first lithography was performed on the thermally oxidized surface of the SOI wafer, and Ti/Pt (30 nm/250 nm) was deposited to form the conductive layer. Thereafter, a SiO_2_ layer with an 800 nm thickness was deposited to establish the insulator area and then patterned and etched to expose all recording sites and bonding pads. Finally, the silicon layer and the upper thermally oxidized layer were selectively deep etched to define the shape of the MEA. After three-layer patterning, the individual electrode was released by wet etching from the back of the SOI and welded on the printed circuit board (Fig. S1).

When the MEA was packaged, we modified the surface of the recording sites with platinum nanoparticles (PtNPs) to reduce the impedance and improve the signal-to-noise ratio^27^. PtNPs were modified in the plating solution by mixing 48 mM H_2_PtCl_6_ and 4.2 mM Pb(CH_3_COO)_2_ at a 1:1 ratio. The working electrode of the MEA and the auxiliary electrode of the platinum filament formed a two-electrode configuration. PtNPs were electrodeposited via chronoamperometry (CA, −1.2 V, 50 s) on an electrochemical workstation (Gamry Instruments, USA).

### Subjects

Two adult male Sprague‒Dawley (SD) rats were used. Before surgery, they were kept in a 12 h on/off light cycle with free access to water but with restricted access to food to keep their weight at ~90% of the normal level. All animals weighed ~350 g at the time of surgery. All experiments were carried out with the permission of Beijing Association on Laboratory Animal Care and approved by the Institutional Animal Care and Use Committee at Aerospace Information Research Institute, Chinese Academy of Science (AIRCAS).

### Craniotomy and MEA implantation

Rats were first deeply anesthetized by inhaling 5% isoflurane and then fixed on a stereotaxic device under 0.5–2% isoflurane to maintain general anesthesia. A 37 °C constant thermal blanket was placed below the rats to keep them at a comfortable temperature. A craniotomy was performed to expose the site for implantation of the MEA (AP: −8.9 mm, ML: 4.3 mm). Before implantation, we fastened the MEA to the shuttle plate of a self-designed microdriver, which can drive the MEA up and down along a set angle according to the shape of MEC (Fig. S2). The minimum step of the microdriver was 30 μm. The MEA was inserted slowly in the sagittal plane with the longest shank pointing anteriorly at the direction of 15° and stopped in the superficial layer of the MEC (DV: 2 mm). Five stainless-steel screws were fixed on the skull connecting to ground wires, serving as ground electrodes and fixed anchors. Then, the shell of the microdriver was secured to the skull with self-curing tooth acrylic, whereas the MEA was covered with Vaseline to maintain its mobility along with the shuttle of the microdriver.

After behavioral tests, we removed the electrodes and reimplanted the MEA coated with the red fluorescent dye DiI (Beyotime, China) at the same location. Then, the rats were anesthetized and perfused with saline and 4% paraformaldehyde. After that, brains were removed and then placed in 20 and 30% sucrose solution for dehydration, and frozen sagittal brain sections of were cut with a thickness of 30 µm. As shown in Fig. S3, the implantation traces of the MEA corresponded to the MEC.

### Behavioral training and testing protocol

Before the MEA implantation surgery, rats were trained to run in an open field arena made from a gray square uncovered acrylic box (120 cm × 120 cm × 60 cm high). When training in the box, crumbs were sprinkled so that they covered the box arena completely, and the rats freely foraged for 30 min per day. After two weeks of training, the rats could run all over the open field without food reward, and then MEA implantation surgery was performed.

After a week of surgical recovery, testing was carried out. The testing protocol consisted of three trials (Fig. S4). For each trial, the tested rat ran freely in the open field, and the implanted MEA was connected to a recording cable. Each trial lasted for nearly 20 min, and the tested rat rested outside the open field for 5 min between trials. Planar, object and social landmarks were used in three trials. The planar landmark was a planar white card (15 cm × 15 cm) on one wall. The object landmark was a white cube (15 cm × 15 cm × 15 cm). For the social landmark, a new rat was placed in a square white box of the same size as the object landmark, and its outward facing side was transparent so that the new rat was visible to the tested rat. After daily tests, the MEA was advanced in steps of 30 µm by the microdriver.

### Neurophysiological data acquisition and behavioral tracking

In each trial, the entorhinal neural activities and behaviors of the tested rats were recorded synchronously. The neuronal signals of the MEC were transmitted via a 16-channel 10 × gain detachable headstage and acquired by the multilevel regulation and high-throughput neural signal detection instrument (AIRCAS-128, China) at 30 kHz^28^. The threshold for signal amplitudes was set to 3 times the noise level. Neural spikes and LFPs were divided by a high-pass filter (>250 Hz) and a low-pass filter (0–250 Hz), respectively. For behavioral tracking, the tested rat’s behaviors were recorded by an overhead optical camera, and the positions, including *X* and *Y* coordinates, were tracked by the behavioral analysis software (EthoVision XT 15, Noldus, China) from the recording video at 30 Hz.

### Identification of grid cells

We used established methods to identify grid cells^29,30^. First, the open field was separated into bins of 1 × 1 cm^2^, and the firing rate was calculated by dividing the counts of spikes by the dwell time of the tested rat in each bin. Then, a firing rate map was obtained by Gaussian smoothing of the spike firing rate for each bin. Second, the autocorrelogram was calculated with values for each bin defined as the Pearson correlation between the original rate map and shifted map along the horizontal and vertical axes. Subsequently, the correlations of a circular region involving six local maximums nearest to the center of the autocorrelogram with its rotations in two groups of degrees (group one: 60° and 120°; group two: 30°, 90°, and 150°) were calculated. The grid score was calculated as the difference between the minimum coefficient of the first group and the maximum coefficient of the second group. Grid cells were defined as cells with a grid score exceeding a threshold of 0.4 according to a previous study^31^.

For each grid cell, the spacing and orientations of the grid pattern were calculated. The spacing was defined as the average of the six distances between the center and near hexagonally arranged peaks in the autocorrelogram. The orientations were defined as three axial counterclockwise angles of the inner hexagonal pattern versus the reference direction of the visual cue.

### Analysis of firing fields

In each session, the firing fields were estimated as connected regions containing at least 30 bins (30 cm^2^) where the firing rate exceeded 20% of the maximum firing rate map. Thereafter, we divided the bins of the open field into three annular zones with equal widths according to the distance with the visual cue (inner zone: 0–52 cm, central zone: 52–93 cm, and outer zone: 93–134 cm). The firing fields were grouped according to the inner zone, central zone, and outer zone.

### Theta modulation analysis

To determine whether LFPs coupled with spikes, LFPs were first bandpass filtered (5–8 Hz), and instantaneous phases were obtained by the Hilbert transform. Then, theta modulation strength was assessed by the Rayleigh test with the null hypothesis of spike phases being equally distributed^32^. The theta modulation was validated with the proportion of the null hypothesis below 0.05.

## Results

### Impedance and stability test of the MEA

**Fig. 2:**
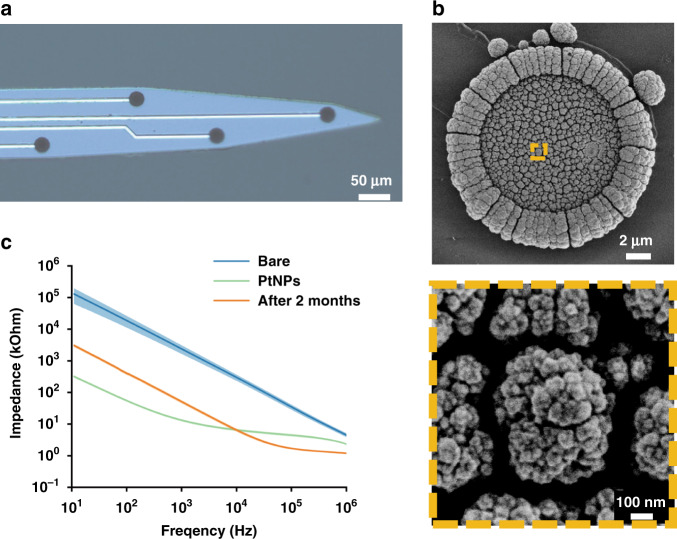
Morphology and electrochemical characterization of PtNP-modified electrodes. **a** Recording sites after modification with PtNPs. **b** SEM images of a single modified site (top) and a local 20× magnification display of PtNPs (bottom). **c** Impedance characteristics at different frequencies of bare, modified microelectrodes and those after 2 months in vivo (*n* = 16)

In our study, the detection sites of the MEA were modified with PtNPs to improve detection sensitivity. The morphology of the PtNP-coated site surface was visualized via an optical microscope (Fig. 2a) and a scanning electron microscope (SEM). It could be observed that PtNPs provided rough and porous structures (Fig. 2b), thus enhancing the specific surface area and improving the conductivity of electrodes. To test the stability of PtNP-modified sites in vivo, the implanted MEA was removed after recording for two months and characterized using electrochemical impedance spectroscopy (EIS) in phosphate buffer solution from 10 Hz to 1 MHz, as shown in Fig. 2c. The spectral curve of the bare site was above that of PtNP-modified and implanted electrodes at all frequencies, whereas differences in the latter two curves were less pronounced. Typically, the mean impedance at 1 kHz was reduced significantly by a factor of 180.3 from 2397.5 ± 183.1 to 13.3 ± 1.2 kΩ when modified with PtNPs and then increased 3.4 times to 44.9 ± 5.8 kΩ for two months in vivo. The results showed that PtNP-modified MEA could provide an effective and stable recording performance for two months.

### Rate remapping induced by a social landmark

**Fig. 3:**
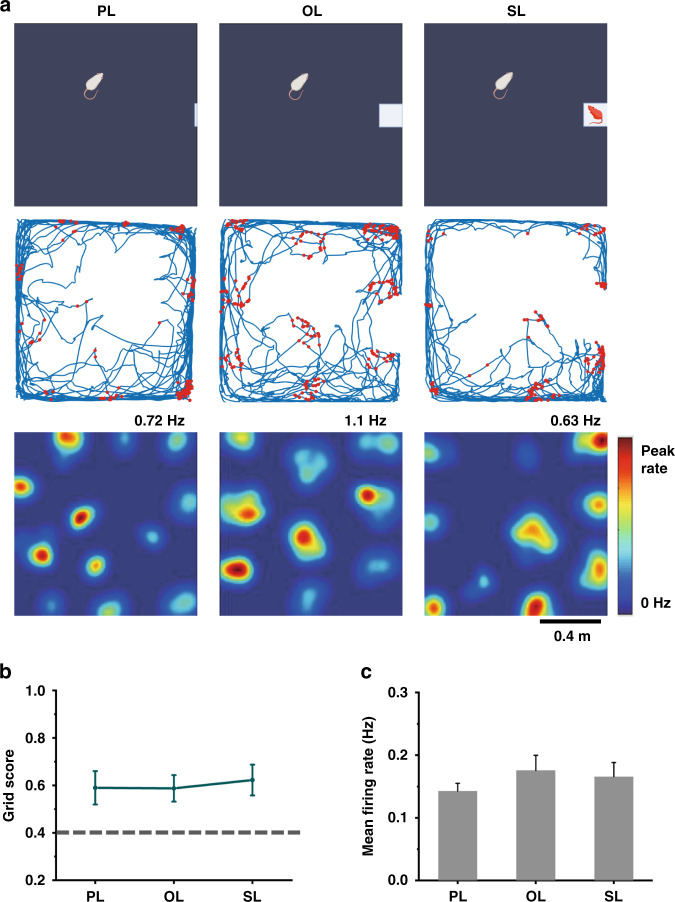
Procedures for grid cell monitoring on exposure to planar, object, and social landmarks. **a** Example of a grid cell shown in trajectory maps with red spike position dots (middle) and firing rate maps (bottom) under three conditions depicted in the schematic diagrams (top). Behavioral schema consisting of three trials, in which rats explored the open field with white planar paper, a 3D object, and a caged rat in turn. **b** Statistics of grid cells with three landmarks revealed that object and social landmarks did not influence the values of grid scores of grid cells (*p* > 0.05, paired t test, *n* = 6). The dashed line indicates the threshold of grid scores, above which the grid cells were identified. **c** No obvious differences in mean firing rates were detected for grid cells under the three conditions (*p* > 0.05, paired t test, *n* = 6)

To determine whether grid cells respond to 3D spatial and social information of visual inputs, the spike activities of the MEC and trajectories of rats were recorded during foraging in the open field with the planar landmark (PL), object landmark (OL), and social landmark (SL) in sequence. Compared with the PL, the OL provides the visual input of 3D spatial information, and the SL provides the visual input of both 3D spatial and social information. In the whole test session containing three trials, stable and consistent spikes of the same neuron were recorded in all periods of trials. (Fig. S5). The grid cells were visualized with spike firing fields forming grid structures covering the whole recording surface. The grid firing patterns for each grid cell were observed from spike-trajectory maps and firing rate maps under three conditions (Fig. 3a), which illustrated the ability of grid cells to maintain spatial representations for novel environments with 3D spatial and social visual inputs. As shown in Fig. 3b, this ability was quantified by calculating grid scores in three trials, and no significant difference was observed (PL: 0.590 ± 0.071, OL: 0.588 ± 0.056, SL: 0.623 ± 0.065, *p* > 0.05, paired t test, *n* = 6). In addition to the spatial symmetry of firing locations, the temporal firing rate was analyzed to ascertain whether OL and SL would influence the discharge activities of grid cells. The variance in the mean firing rates in the three trials was not obvious, as shown in Fig. 3c (PL: 0.143 ± 0.012 Hz, OL: 0.176 ± 0.024 Hz, SL: 0.166 ± 0.022 Hz, *p* > 0.05, paired t test, *n* = 6). The results demonstrated that OL and SL had almost no effect on the spatial periodicity and temporal stability of firing in MEC grid cells.

**Fig. 4:**
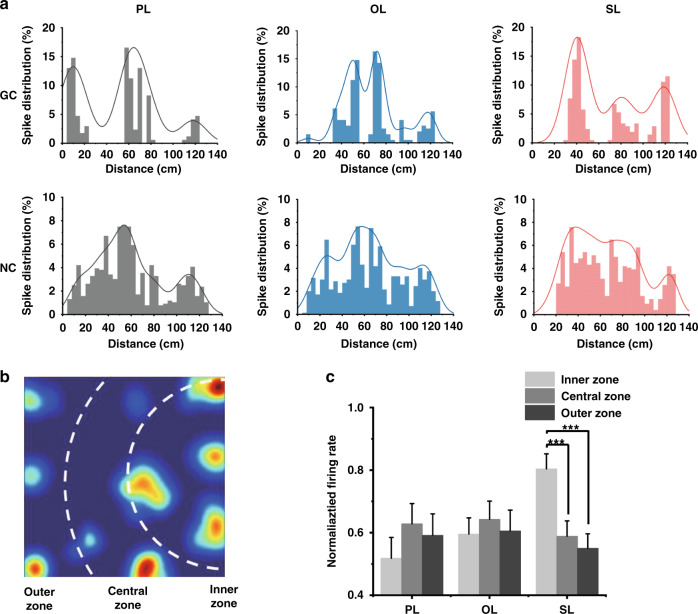
Rate remapping induced by the social landmark. **a** Spike distributions and kernel smoothing density estimates of a typical grid cell (GC) and nongrid cell (NC) in three trials with PL, OL, and SL. **b** A typical firing rate map in a trial with the social landmark. Dashed white curves are boundaries that divided the arena equally according to the distance to the landmarks. **c** The normalized firing rate of each zone in all six grid cells was calculated as the average of the normalized peak firing rate of firing fields in each zone for three landmarks (***p < 0.001, unpaired t test)

Since the overall firing properties of grid cells both in spatial and temporal aspects remain stable, the influence of OL and SL may focus on local firing fields. To determine this, the spike distribution of their distances to the central position of landmarks both in the grid cells and nongrid cells was calculated as shown in Fig. 4a. Two main observations were made according to vertical and transverse comparisons. First, the spike distributions versus distances of the grid cells were discontinuous, whereas those of the nongrid cells were continuous. Kernel smooth density curves of the grid cells revealed three or four clear peaks in contrast to the unimodal distribution of the nongrid cells. The former was derived from the hexagonal arrangement of firing fields that layered around the landmark, and the latter approximated normal distributions of random distances of nonspatial tuned spikes. Second, a key observation of SL was that the spike distribution peak nearest to the origin was obviously higher than further peaks, which suggested rate responses of grid cells when rats got close to the SL. Another change occurred both in grid cells with the OL and SL that peak distances were offset compared to the PL, implying the remapping of firing locations, which will be elaborated upon further in the next paragraph. To quantify the rate change of the SL, we calculated the peak firing rate of each firing field for all grid cells and analyzed the relationship with distance to the landmark. For each trial with spikes forming the grid firing pattern, we divided the firing fields into three groups corresponding to three regions in the open field, inner zone, central zone, and outer zone, which gradually increase in distance from the landmarks (Fig. 4b). Then, the average normalized firing rate of grouped firing fields in each zone was calculated to examine the influence on the firing rate by distance to landmarks. As shown in Fig. 4c, under social input, the normalized firing rate of inner firing fields was significantly higher than those of central and outer firing fields (inner zone: 0.804 ± 0.048, central zone: 0.589 ± 0.049, and outer zone: 0.550 ± 0.046, *p* < 0.0001, unpaired t test); however, this was not observed under planar and object landmarks. This result suggested that social information provokes pronounced rate changes in firing fields in grid cells, namely, ‘rate remapping’.

### Global remapping induced by both object and social landmarks

**Fig. 5:**
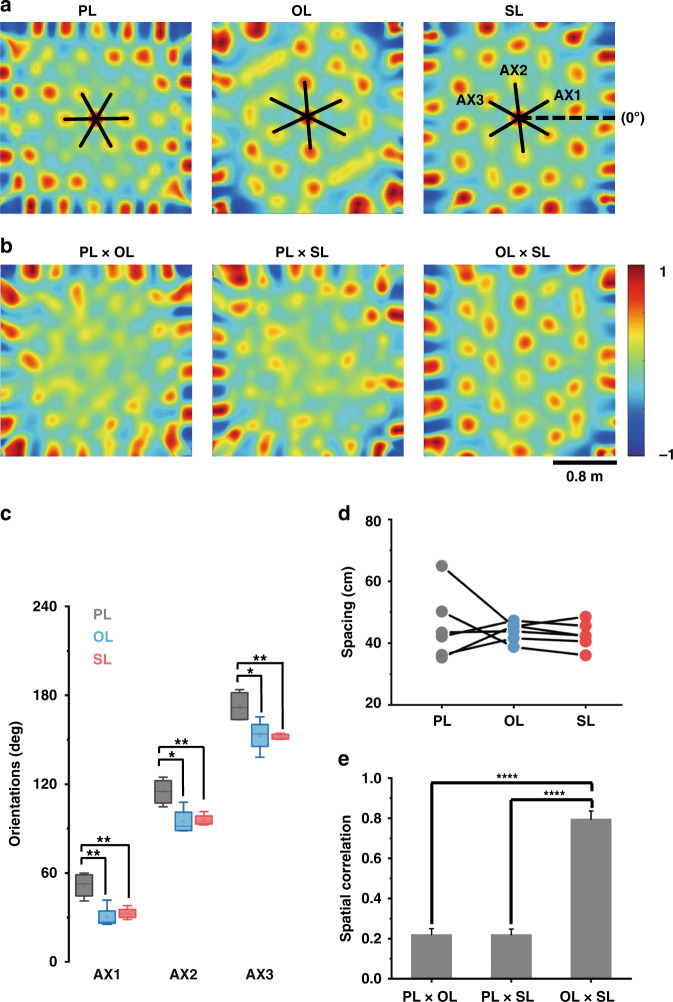
Global remapping induced by 3D landmarks containing an OL and SL. **a** Grid pattern of the spatial autocorrelogram rotates and scales in response to the object and social stimulus. Black solid lines and dashed lines indicate grid axes (AX) in three directions and horizontal reference (0°), respectively. **b** Cross-correlation maps of grid firing rate matrices for two of the corresponding three trials in (**a**). **c** Boxplot showing orientation distribution for three axes of all cells from one rat in three trials (*p < 0.05, **p < 0.01, paired t test, *n* = 4; center lines, individual medians; boxes, 25th–75th percentile range; whiskers, extreme value). **d** Statistics of grid spacing responding to three landmarks (*n* = 6). **e** Spatial correlation calculated for two spatial autocorrelograms of the innermost hexagonal region under three conditions. (****p < 0.0001, paired t test, *n* = 6)

In addition to the rate changes evoked by the SL, the locations of firing fields were remapped in the OL and SL conditions. The grid pattern of firing fields can be represented by the central peak and six near equidistant peaks in the autocorrelogram (Fig. 5a). When replacing the PL with an OL or SL with the same 3D size, grid patterns of autocorrelograms were remapped, such as by rotation and scaling—so called ‘global remapping’, which was not reproduced by changing from an OL to a SL. The distinction between PL and OL/SL and the similarity between OL and SL can be validated by the cross-correlogram between them (Fig. 5b). The apparent patterns in the cross-correlogram of OL × SL revealed similar spatial structures of firing fields, whereas the blurry peaks in the other two maps revealed different firing ensembles between PL and 3D landmarks. More specifically, the orientations and spacing of grid patterns in autocorrelograms for each grid cell were measured to examine the consistency of geometric structure during the three trials. The orientations of grid cells of one rat in the same environment were clustered on the similar axis, especially one axis in the PL condition, which was almost parallel to one of the walls (172.7 ± 5.3°, *n* = 4). The statistics of grid orientations of one rat exhibited a significant change from PL to OL/SL conditions and no significant difference between OL and SL conditions (Fig. 5c), which indicated the rotation of grid patterns in an environment with 3D landmarks. A similar result in another rat is shown in Fig. S7. In addition to orientations, Fig. 5d illustrates that spacing varied obviously between PL to OL/SL conditions, either larger or smaller, demonstrating scaling up or scaling down. In contrast, the spacing between OL and SL exhibited less variation. Furthermore, the grid patterns between OL and SL showed a significantly higher spatial correlation than the others, as shown in Fig. 5e (PL × OL: 0.219 ± 0.031, PL × SL: 0.206 ± 0.028, OL × SL: 0.796 ± 0.041, *p* < 0.0001, paired t test, *n* = 6). In conclusion, the grid pattern rotation and scaling from planar to 3D landmarks and maintenance between OL and SL suggested that global remapping would be evoked by the 3D spatial information rather than social information in the environment.

### Theta modulation under three landmarks

**Fig. 6:**
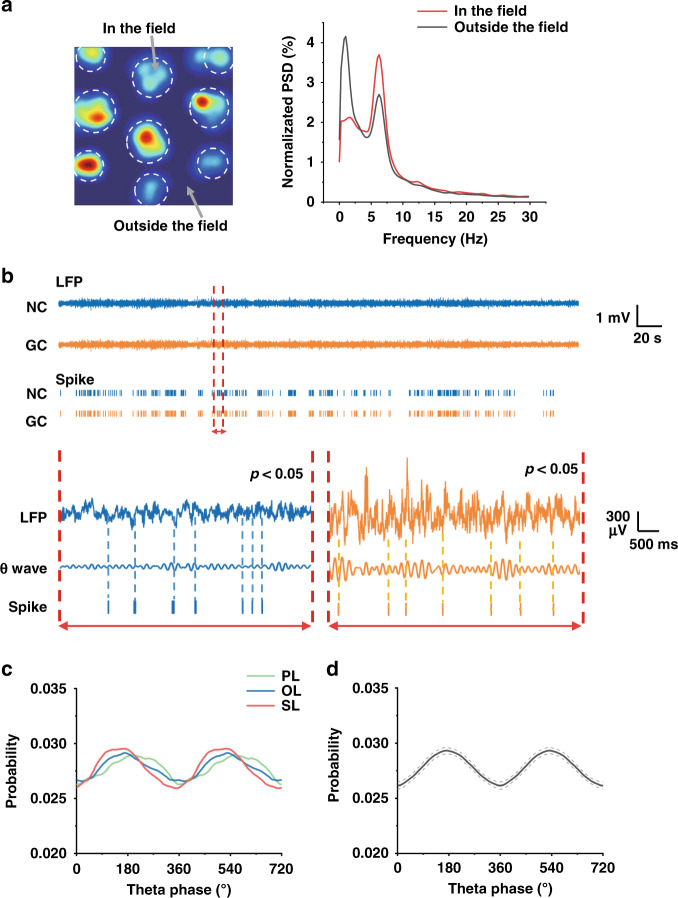
Theta oscillations and phase locking in MEC neurons under three landmark conditions. **a** Dashed white circles in the firing rate map indicate the areas of firing fields, dividing the activities of rats into the two groups of ‘in the field’ and ‘out the field’ segments (left). The averaged normalized PSD (%) of the LFPs of ‘in the field’ and ‘out the field’ segments revealed the obviously high power of the theta band (5–8 Hz) in the firing fields (right). **b** Top: the synchronous LFPs and spikes of a typical grid cell (GC) and nongrid cell (NC) recorded by an MEA in the trial shown in (**a**). Bottom: a piece of LFPs and spikes of NC (left) and GC (right) indicated in the top signals. Middle waves between LFPs and spikes are theta waves filtered in the 5–8 Hz range. Tick marks of spike timestamps are locked in troughs of LFPs and theta waves (*p* < 0.05, Rayleigh test). **c** Spike probability of a typical grid cell in three trials with PL, OL, and SL as a function of theta phase. **d** Averaged spike probability of all grid cells in all trials as a function of theta phase. Dotted lines indicate the standard error of the mean (*n* = 18)

Theta modulation is a crucial mechanism of spatial memory in population neurons, and previous studies have shown that theta oscillations are present in the activities of grid cells^19,32^. To explore the relationship between the theta rhythm of LFPs and spikes of grid cells, the LFPs when rats ran in the spike firing fields and outside the fields were separately analyzed. First, we divided the recording session into in-the-field and outside-the-field segments, and time-weighted averages of normalized power spectral density (PSD) for each segment were calculated, as shown in Fig. 6a. The results showed that when rats were in the firing fields, LFPs were dominated by the frequency range from 5 to 8 Hz in the theta band, revealing synchronous activities with theta-dominated LFPs and spikes of grid cells. In contrast, the normalized PSD of outside-the-field segments had a global peak in the delta band of 1–4 Hz and a similar frequency band but a lower local peak with in-the-field segments, which may be derived from the confusion in the division of the two kinds of segments. To further illustrate the relationship between spikes and the theta band of LFPs, the instant phase of spike timestamps in LFPs were extracted and observed to be locked to troughs of theta oscillation (Fig. 6b). Both grid cells and nongrid cells exhibited theta phase locking. In addition, Rayleigh tests were performed to assess theta phase locking (*p* < 0.05). Then, we asked whether theta modulation strength would be influenced by different landmarks. The spike probability of grid cells under three landmarks revealed a similar theta phase distribution with their peaks at 180° and troughs at 0° (Figs. 6c, d and S8), proving the phase locking in troughs of theta rhythm. In conclusion, our observations illustrated the general evidence of phase locking in MEC neurons and the small effect of 3D spatial and social information on theta modulation strength.

## Discussion

In the present study, the fabricated MEA was proven to be able to detect grid cells. The structural design of stepped shanks enabled electrophysiological detection covering the region of the MEC while reducing damage to other brain regions. Furthermore, the designed arrangement of recording sites and the axial movement controlled by the self-designed microdriver provide an estimate for detecting locations, which addressed the advantages of implantable MEA in exploring the grid cells in the MEC for freely moving rodents. The PtNP-modified sites had a stable detection performance over a period of two months. The change in impedance after two months may account for two reasons. First, tiny movements between the electrodes and brain tissues caused friction on the surface of the detection sites, which promoted delamination of the PtNPs. Second, inflammatory compounds induced by tissue injury adhered to the surface of detection sites, affecting the interface characteristics of electrodes. Future work is needed to explore the novel decoration of nanomaterials to improve biocompatibility.

Our study provided evidence for a mechanism by which hexagonal firing patterns of grid cells could be rotated and scaled by the 3D spatial features of the environment. Previous works have demonstrated that grid cell patterns with one axis are almost parallel to one of the walls of a square arena^33,34^. Our results also confirmed this but illustrated that orientations of the hexagonal structure would be shifted with 3D landmarks in the square environment. In comparison with the traditional planar visual cue, the 3D landmarks not only provided a different visual input for rats but also changed the landform surface of the square environment, thereby affecting the rats’ planned actions with new reference frames. The invariant grid firing patterns when replacing the object with a novel rat in the cage of the same size elucidated that grid patterns were related to the 3D geometric features of landmarks rather than the social information, which consolidated that 3D landmarks induced global remapping by altering the topography of the environment.

Grid cells exhibited rate remapping when transforming the object to a social landmark, in which condition grid fields that were closer to the landmark expressed a higher firing rate in each trial. This observation reminded us of previous reports of social-remapping place cells in the hippocampus, which would generate new firing fields closer to the novel animal^35^. Because hippocampal neurons are anatomically^36^ and physiologically^15,37^ associated with those in the MEC, the similar response to social inputs of grid cells and place cells may suggest that social information is conveyed through the entorhinal-hippocampus neural circuit. Previous reports^38,39^ illustrated that the lateral and medial entorhinal cortices processed nonspatial information and spatial information, respectively, and transformed them into the hippocampus. Regarding social information, the lateral entorhinal cortex was thought to provide a stronger direct excitatory drive to the hippocampus than the MEC^12^, which agrees with our results of a stable temporal firing rate and consistent firing patterns during trials with object and social landmarks. However, our observation of rate remapping demonstrated that grid cells in the MEC responded to social information in a more sophisticated, spatially relevant way. This finding suggested a causal relationship between the MEC and hippocampus during animals’ social activities. Although grid cells represented both spatial and social features in firing patterns, it was unclear whether the MEC served as the preprocessing node of social information prior to transmission to the hippocampus. Since firing patterns of grid cells require hippocampal back-projections^40^, it is also possible that rate remapping is evoked by conveying social information from the hippocampus to the MEC. Future work is needed to explore the propagation mechanism of social information by the simultaneous recording of entorhinal grid cells and hippocampal place cells during social activities.

## Conclusions

In this study, an MEC-shaped MEA was fabricated for the detection of variations in grid cell activities when rats foraged in the open field under planar, 3D object, and social landmarks in sequence. The results showed that 3D landmarks induced global remapping of grid firing fields, including the variation of grid spacing and orientations. However, grid patterns remained coherent between object and social landmarks with the same size, whereas rate remapping was observed under the social condition that firing fields closer to the landmark exhibited a higher firing rate. In addition, theta oscillations appeared in the grid firing fields, and theta-frequency phase locking was maintained under three landmarks, which proved that theta modulation was a common population discharge mechanism to regulate the spatial representations of grid cells. Our study supported the pattern separation mechanism that grid cells distinctly represent the spatial and social information by the grid patterns and firing rate distribution.

## Supplementary information

Supplementary Information video

Supplemental Material
