# Supplementary material for: Grid cell remapping under three-dimensional object and social landmarks detected by implantable microelectrode arrays for the medial entorhinal cortex
Source: Microsyst Nanoeng. 2022 Sep 16;8:104. doi: 10.1038/s41378-022-00436-5 (PMC9481550; doi:10.1038/s41378-022-00436-5)
Supplement: Supplementary file 2 — Supplemental Material [file 41378_2022_436_MOESM2_ESM.docx]

**Supplementary Information**

Grid cell remapping under three-dimensional object and social landmarks detected by implantable microelectrode arrays for medial entorhinal cortex

Zhaojie Xu^1,2^, Fan Mo^1,2^, Gucheng Yang^1,2^ Penghui Fan^1,2^, Yiding Wang^1,2^, Botao Lu^1,2^, Jingyu Xie^1,2^, Yuchuan Dai^1,2^, Yilin Song^1,2^, Enhui He^1,2^, Shihong Xu^1,2^, Juntao Liu^1,2^, Mixia Wang^1,2^ and Xinxia Cai ^[[1]](#footnote-1),^^[[2]](#footnote-2)^

**This file includes:**

Figures S1–S8.

Movie S1

*
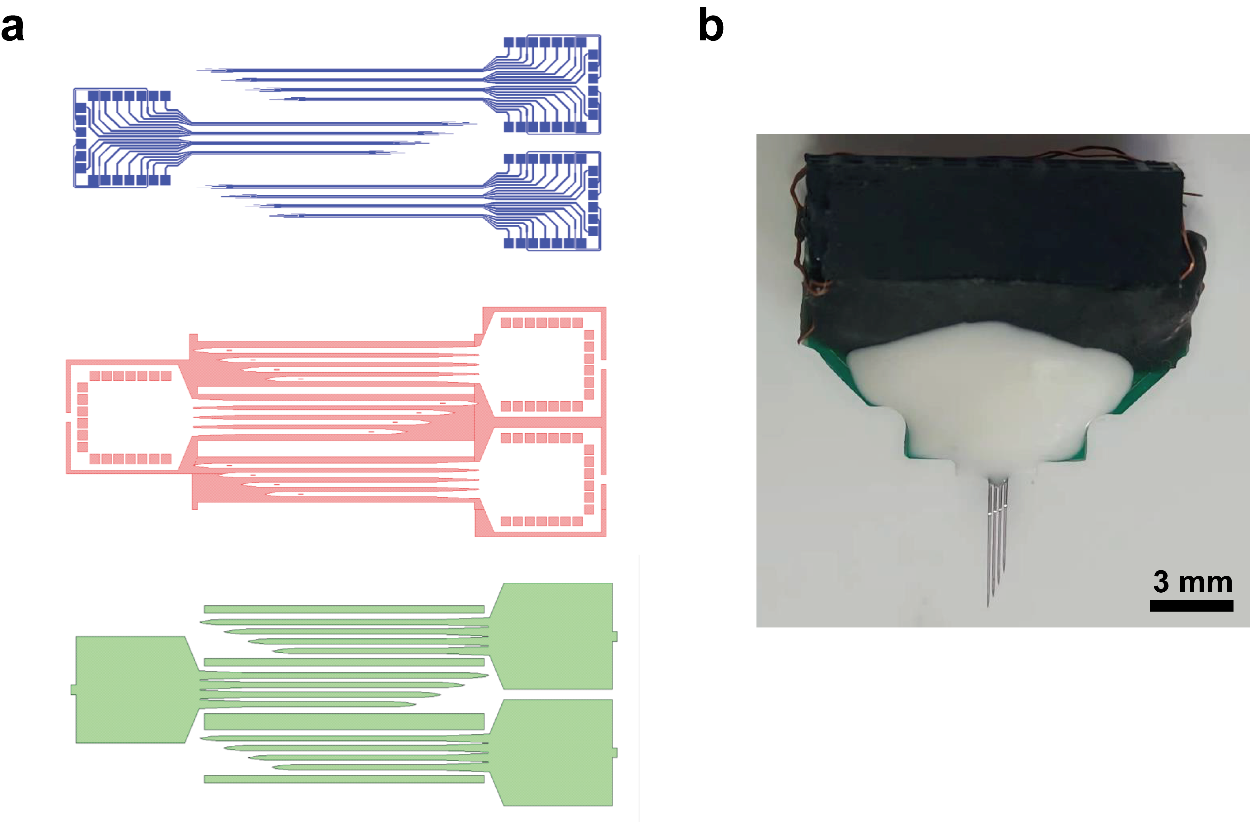
***Figure S1**

**Fig. S1 Mask design and individual packaged product of MEA.** **a** Three layer masks of MEA. Top: conductive layer mask, middle: insulating layer mask, bottom: MEA shape mask. **b** Prepared and packaged MEA.

**Figure S2**

***
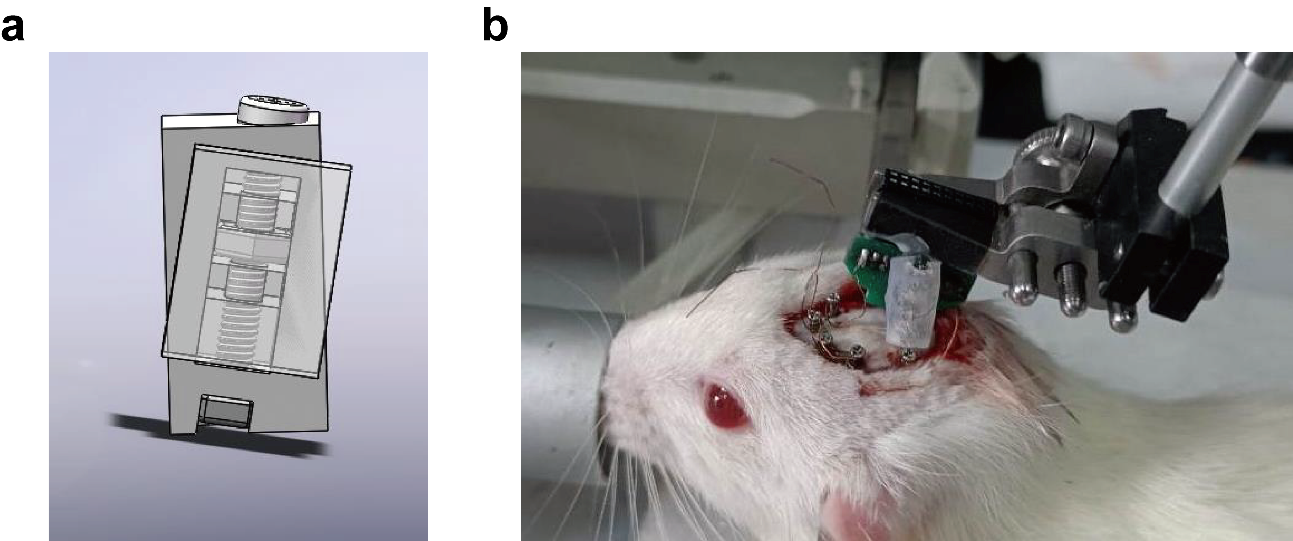
***

**Fig. S2 Illustration of self-designed micro driver and its application in the surgery.** **a** Micro driver contains a shell and shuttle screwed by a screw. The shuttle is fasten with MEA and move at the set direction of 15°. **b** A 3D-printed micro driver used in a surgery of MEA implantation.

**Figure S3**

**
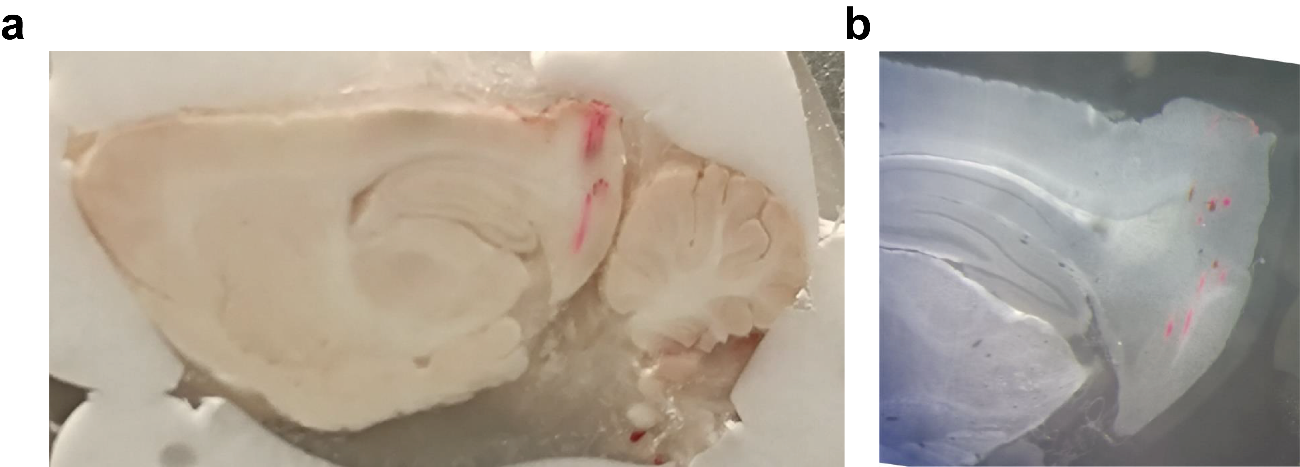
**

**Fig. S3 Postmortem histochemistry. a** Red traces indicating the implanted MEA were in MEC of the brain. **b** A 30 µm thickness sagittal brain section with the traces of MEA shanks.

**Figure S4**

**
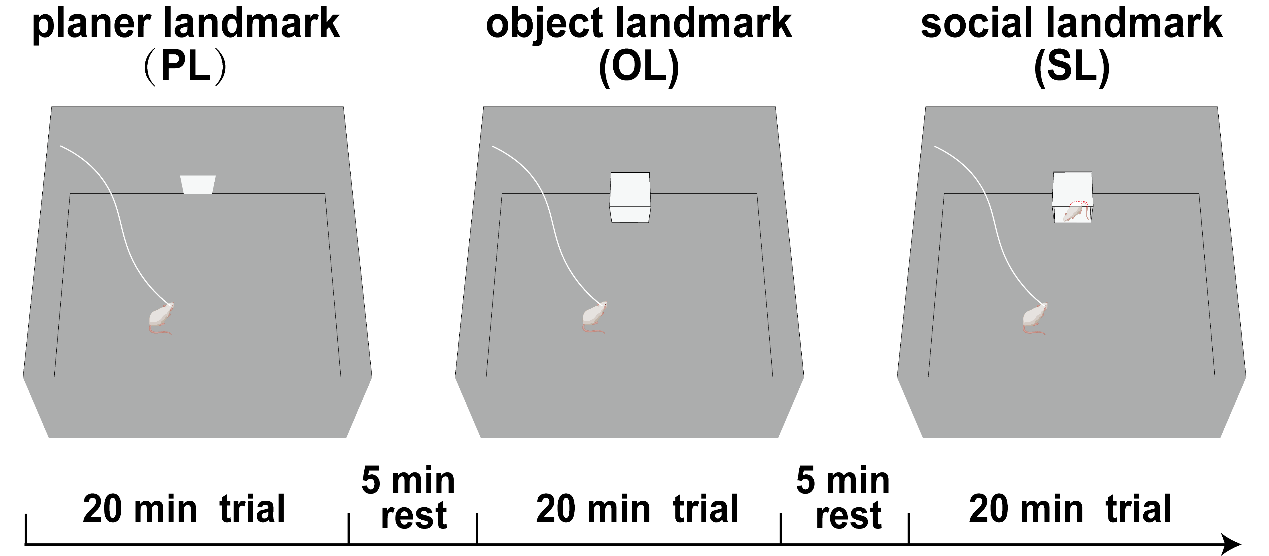
**

**Fig. S4 The testing protocol consisted of three trials with the planer, object and social　landmarks respectively.**

**Figure S5**


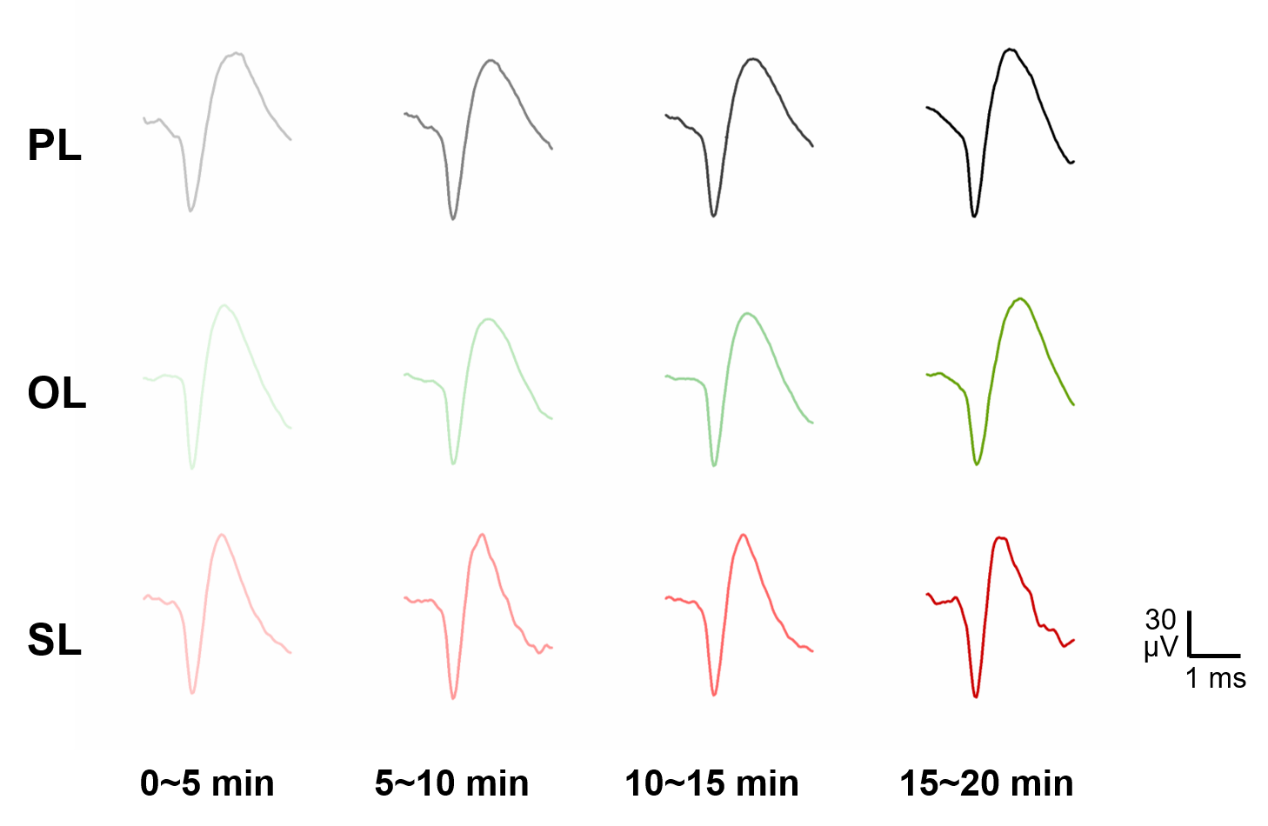


**Fig.S5 The mean spike waveforms of a typical grid cell under PL, OL and SL conditions in four periods:0~5 min, 5~10 min, 10~ 15 min, 15~ 20 min.**

**Figure S6**


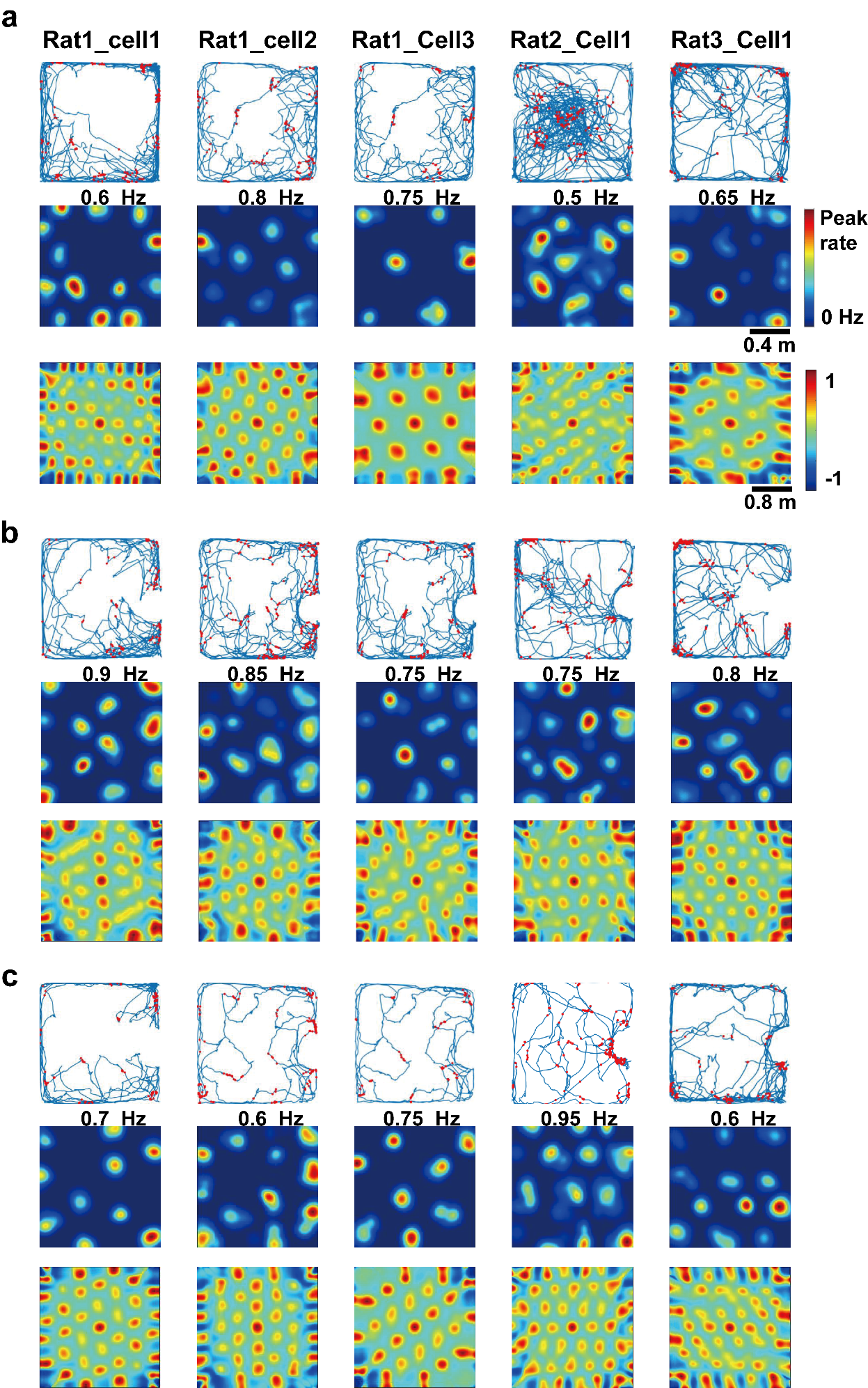


**Fig. S6 Display of all detected grid cells excepted one shown in Fig. 3. a** Spike-on-trajectory maps (top), firing rate maps (middle) and autocorrelograms (bottom) under PL condition. **b, c** same graphs with **a** but under OL and SL conditions.

**Figure S7**

***
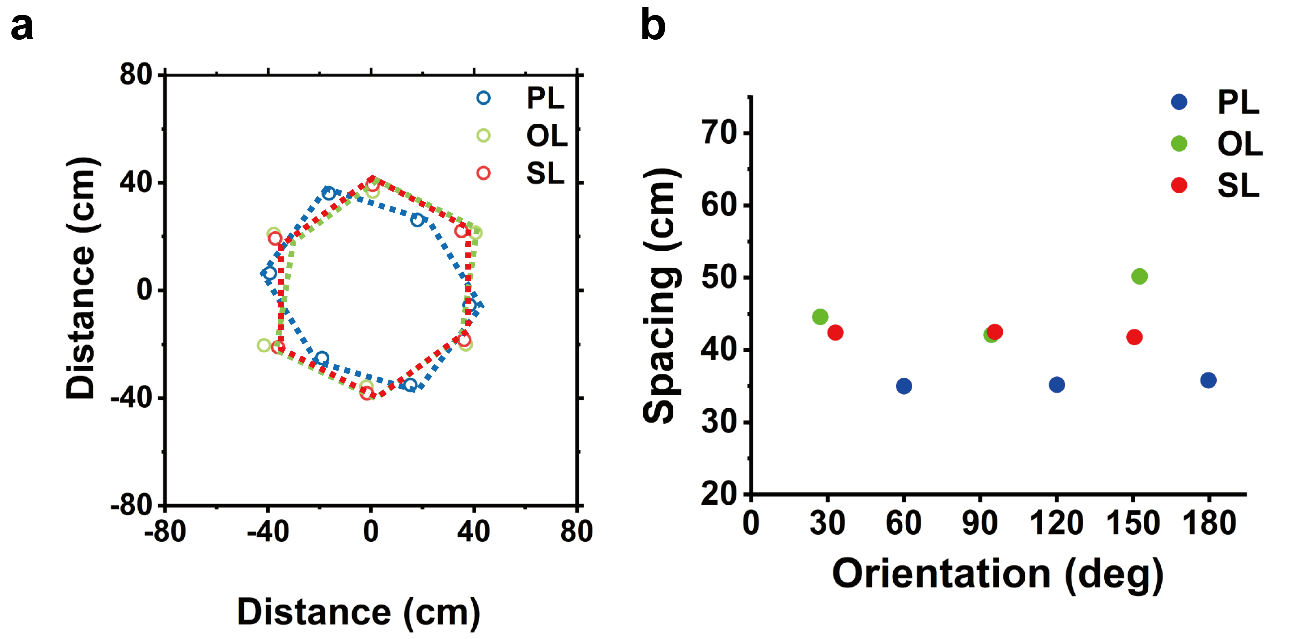
***

**Fig. S7 Global remapping of a typical grid cell exhibited in the change of grid firing patterns containing spacing and orientations. a** Grid firing patterns formed by six equidistant peaks closest to the center of autocorrelogram. The pattern of PL rotates and scales in OL and SL. **b** Scatter plot of spacing and orientations of grid firing patterns shown in **a** for three axes. Both spacing and orientations are close between OL and SL, which are distant from those in PL.

**Figure S8**

*
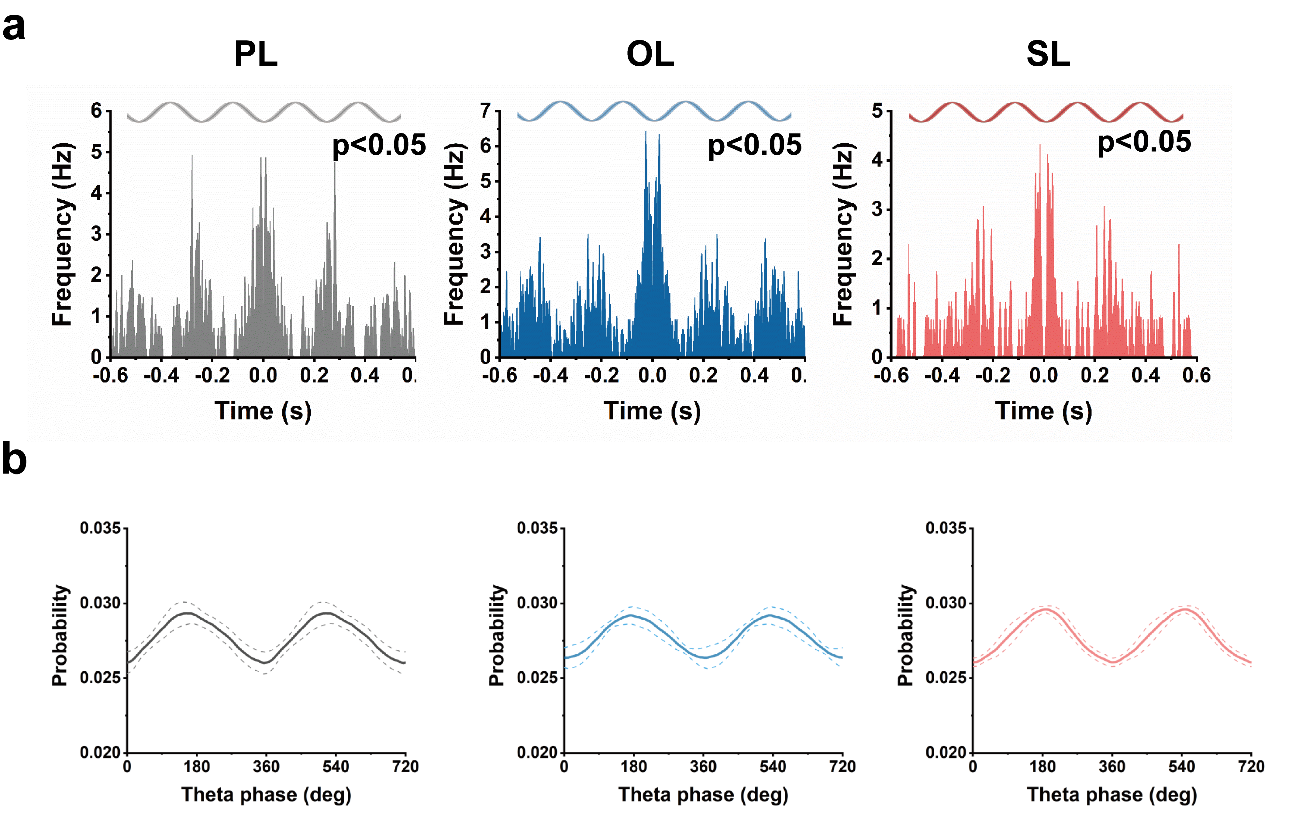
*

**Fig. S8 Theta modulation of grid cells under three landmarks.** **a** Spike time autocorrelations which could be fit with sine wave function of 5 Hz, showing the common evidence of theta modulation in three landmarks. (p < 0.05, Rayleigh test). **b** Averaged spike probabilities of grid cells in trials with PL (n = 6), OL (n =6) and SL (n = 6) as a function of theta phase. Dotted lines indicated the standard error of mean.

1. Correspondence: Mixia Wang (wangmixia@mail.ie.ac.cn) or Xinxia Cai (xxcai@mail.ie.ac.cn)

   State Key Laboratory of Transducer Technology, Aerospace Information Research Institute, Chinese Academy of Sciences, Beijing 100190, China [↑](#footnote-ref-1)
2. University of Chinese Academy of Sciences, Beijing 100049, China [↑](#footnote-ref-2)
